# *Ex vivo* tetramer staining and cell surface phenotyping for early activation markers CD38 and HLA-DR to enumerate and characterize malaria antigen-specific CD8^+^ T-cells induced in human volunteers immunized with a *Plasmodium falciparum* adenovirus-vectored malaria vaccine expressing AMA1

**DOI:** 10.1186/1475-2875-12-376

**Published:** 2013-10-29

**Authors:** Robert Schwenk, Glenna Banania, Judy Epstein, Yohan Kim, Bjoern Peters, Maria Belmonte, Harini Ganeshan, Jun Huang, Sharina Reyes, Anette Stryhn, Christian F Ockenhouse, Soren Buus, Thomas L Richie, Martha Sedegah

**Affiliations:** 1 Malaria Vaccine Branch, Military Malaria Research Program, Walter Reed Army Institute of Research, 503 Robert Grant Avenue, Silver Spring, MD, USA; 2 Malaria Department, Infectious Disease Directorate, Naval Medical Research Center, 503 Robert Grant Avenue, Silver Spring, MD 20910, USA; 3 Laboratory of Experimental Immunology, University of Copenhagen, Copenhagen, Denmark; 4 La Jolla Institute for Allergy and Immunology, La Jolla, CA, USA

**Keywords:** Malaria, CD8+ T cells, MHC-I tetramers, Activation markers

## Abstract

**Background:**

Malaria is responsible for up to a 600,000 deaths per year; conveying an urgent need for the development of a malaria vaccine. Studies with whole sporozoite vaccines in mice and non-human primates have shown that sporozoite-induced CD8^+^ T cells targeting liver stage antigens can mediate sterile protection. There is a need for a direct method to identify and phenotype malaria vaccine-induced CD8^+^ T cells in humans.

**Methods:**

Fluorochrome-labelled tetramers consisting of appropriate MHC class I molecules in complex with predicted binding peptides derived from *Plasmodium falciparum* AMA-1 were used to label *ex vivo* AMA-1 epitope specific CD8^+^ T cells from research subjects responding strongly to immunization with the NMRC-M3V-Ad-PfCA (adenovirus-vectored) malaria vaccine. The identification of these CD8^+^ T cells on the basis of their expression of early activation markers was also investigated.

**Results:**

Analyses by flow cytometry demonstrated that two of the six tetramers tested: TLDEMRHFY: HLA-A*01:01 and NEVVVKEEY: HLA-B*18:01, labelled tetramer-specific CD8^+^ T cells from two HLA-A*01:01 volunteers and one HLA-B*18:01 volunteer, respectively. By contrast, post-immune CD8^+^ T cells from all six of the immunized volunteers exhibited enhanced expression of the CD38 and HLA-DR^hi^ early activation markers. For the three volunteers with positive tetramer staining, the early activation phenotype positive cells included essentially all of the tetramer positive, malaria epitope- specific CD8^+^ T cells suggesting that the early activation phenotype could identify all malaria vaccine-induced CD8^+^ T cells without prior knowledge of their exact epitope specificity.

**Conclusions:**

The results demonstrated that class I tetramers can identify *ex vivo* malaria vaccine antigen-specific CD8^+^ T cells and could therefore be used to determine their frequency, cell surface phenotype and transcription factor usage. The results also demonstrated that vaccine antigen-specific CD8^+^ T cells could be identified by activation markers without prior knowledge of their antigen-specificity, using a subunit vaccine for proof-of-concept. Whether, whole parasite or adjuvanted protein vaccines will also induce {CD38 and HLA-DR^hi^}^+^ CD8^+^ T cell populations reflective of the antigen-specific response will the subject of future investigations.

## Background

Malaria is responsible for up to 600,000 deaths per year, indicating the urgency to develop a malaria vaccine. Immunization with whole sporozoite (spz) vaccines can confer sterile immunity to mice [1], non-human primates [2] and humans [3] and in the case of mice [4] and non-human primates [5] CD8^+^ T cells against pre-erythrocytic stages of malaria appear to be *sine qua non* mediators of protection. Recent data has also shown that sterile protection induced in humans with a subunit-adenovirus-vectored vaccine significantly associates with the presence of CD8^+^ T cells [6]. CD8^+^ T cells are thought to confer protection, at least in part, by means of cytokine-mediated inhibition of intra-hepatic parasite development [7,8]. Therefore, it has become common practice to attempt to correlate vaccine-induced CD8^+^ T cell cytokine production with protective immunity. Characterization of malaria antigen-specific CD8^+^ T cells by this method is limited, however, as their effector function might be due to cytokines distinct from those being measured (for example, by flow cytometry), or to non-cytokine-related mechanisms such as Fas/FasL-mediated induction of apoptosis or the direct killing of hepatocytes through release of perforin and granzyme. There is also the requirement to re-stimulate the cells *in vitro* with their specific antigen, which in the case of whole sporozoite vaccines may not be known. Restimulation could also distort accurate phenotyping of the CD8^+^ T cells by inducing phenotypic changes in the cells. Therefore, as has become clear from animal models, the accurate characterization of CD8^+^ T cells, as they pass through the expansion, contraction and memory phases, into short-lived (terminal) effector cells (SLECs), memory potential effector cells (MPECs), effector memory CD8^+^ T cells (T_EM_), central memory CD8^+^ T cells (T_CM_) or resident memory CD8^+^ T cells, would be better accomplished by direct *ex vivo* examination, either by direct tetramer staining of the cells or by the use of transgenic T cells. Such approaches would also likely enhance the probability of discovering the immunological correlates of this protection.

Direct *ex vivo* identification of human malaria-vaccine antigen-specific CD8^+^ T cells, by labelling with cognate tetramers expressing class I- restricted vaccine antigen epitopes, should be feasible for CD8^+^ T cells induced by subunit vaccines expressing a known malaria antigen, with the proviso that there could be greater than one T cell epitope per antigen and, in the case of a vaccine trial, one would have to take into account the class I genetic diversity of the cohort of volunteers. However, immunization with multistage multi-antigen malaria vaccines, or whole parasite vaccines, could lead to the induction of CD8^+^ T cells against a large number of and, in many cases, potentially uncharacterized epitopes necessitating a different approach for these types of vaccines. Recently, Miller *et al.*[9] demonstrated that the total human CD8^+^ T cell response to yellow fever virus and vaccinia virus could be estimated by identifying all CD8^+^ T cells expressing both the CD38 activation marker and high levels of HLA-DR. Whether a similar approach would be applicable to malaria-specific CD8^+^ T cells remains to be investigated. Consequently, the feasibility of directly identifying malaria antigen-specific CD8^+^ T cells *ex vivo* by the expression of early activation markers was examined. Research subjects immunized with an adenovirus-vectored subunit vaccine inducing strong CD8^+^ T cell responses to known malaria antigens were studied and their antigen-specific CD8^+^ T cell responses in the periphery were quantified by tetramer labelling. In addition, it was also demonstrated that tetramer-positive CD8^+^ T cells from volunteers where tetramer labelling was successful were concordant with those showing the {CD38 and HLA-DR^hi^} ^+^ activation phenotype.

## Methods

### Vaccination protocol

The NMRC-M3V-Ad-PfCA (AdCA) vaccine used in this study is a combination of two separate recombinant adenovirus 5 constructs, one expressing full length *P. falciparum* circumsporozoite protein (CSP) (minus 16 repeats, and insertion of 23 amino acids derived from the 3′-noncoding bovine growth hormone polyadenylation sequence at the C-terminus) and the other expressing full length *P. falciparum* apical membrane antigen-1 (AMA1) (both strain 3D7). Volunteers were administered one intramuscular injection of 2×10^10^ particle units (pu) of the combination vaccine (protocol NMRC.2006.0001), sufficient to induce strong CD8^+^ T cell responses in a majority of study subjects [10,11]. Six subjects were selected from among 18 receiving the vaccine due to (1) known strong antigen-specific responses to the vaccine and (2) availability of frozen peripheral blood mononuclear cells (PBMC). PBMC were not available from any additional research subjects, limiting the sample size of our investigation.

### Collection, isolation and storage of peripheral blood mononuclear cells

PBMC were sampled at pre-vaccination and at day 22/23 post vaccination and were separated from whole blood on Ficoll-Paque and then cryopreserved in liquid nitrogen until used.

### Enrichment of CD8 ^+^ T cells

Because the frequency of antigen-specific CD8^+^ T cells can be as low as one in 100,000, highly enriched CD8^+^ T cell populations were isolated from the subjects’ PBMC. Briefly, PBMC were suspended in MACS buffer (phosphate buffered saline supplemented with 0.5% bovine serum albumin and 2 mM EDTA) and CD8^+^ T cells were isolated by negative magnetic selection on an LS column (Miltenyi Biotec, Auburn, CA) using a CD8^+^ T cell isolation kit (Miltenyi Biotec) according to the manufacturer’s instructions.

### Tetramers

Peptides used for tetramer formation corresponded to class I-restricted epitopes of AMA1 protein that were down-selected as described previously [12]. Peptide-HLA class I tetramers were generated as previously described [13]. Briefly, fully biotinylated denatured recombinant HLA class I molecules were diluted into an excess of recombinant human beta-2 microglobulin and an excess of peptide in question, and incubated at 18°C for 48 hr allowing for folding and complex formation. Then fluorescence labelled streptavidin was added in 4:1 (peptide/HLA complex:Streptavidin) molar ratio to effect tetramerization. The tetramers used in this study along with their predicted epitope binding affinity (IC_50_ nM) are shown in Table 1.

**Table 1:** AMA1-based tetramers used in this study

		**Tetramer tested**				
**Volunteer**	**Volunteer HLA**	**Tetramer HLA**	**AMA-1 epitope**	**IC**_**50 **_**nM**^**1**^	**AMA1 pool**	**sfc/mill to pool**^**4**^
119	A*30:02^2^	A*01:01	TLDEMRHFY	36	Ap4	266
		A*30:02	RYKSHGKGY^3^	201	Ap8	547
125	A*02:01	A*02:01	TQKCEIFNV	658	Ap8	81
126	A*01:01	A*01:01	TLDEMRHFY	17	Ap4	228
		A*30:02	RYKSHGKGY	172	Ap8	153
128	A*02:01	A*02:01	TQKCEIFNV	658	Ap8	31
	B*18:01	B*18:01	NEVVVKEEY	12	Ap10	215
		B*18:01	YEYPLHQEH	26	Ap1	71
		B*18:01	NEFPAIDLF	9	Ap7	99
147	A*30:01	A*30:02	RYKSHGKGY	172	Ap8	13
184	A*01:01	A*01:01	TLDEMRHFY	17	Ap4	568

^1^IC_50_ nM values for the volunteer’s HLA type were predicted using NetMHC web-based software [14].

^2^A*30:02 is a member of the A01 supertype.

^3^The predicted epitope = HGKGYNWGN.

^4^ELISpot response to parent AMA1 peptide pool containing the epitope.

### Flow cytometry reagents

V-500 anti-CD3, V-450 anti-CD4, FITC anti-CD8 and Alexa-700 anti-CD38 were obtained from BD Biosciences (San Jose, CA). Per-CP anti-HLA-DR was purchased from R and D Systems (Minneapolis, MN) and the UV viability dye was acquired from Invitrogen (Grand Island, NY). Human Fc block was from Miltenyi Biotec.

### Labelling of CD8^+^ T cells

Purified CD8^+^ T cells (0.5 to 1 × 10^6^) were re-suspended in approximately 50 ul of MACS buffer in a conventional FACS tube; 5 ul of Fc blocking reagent was added per tube and the cells were placed on ice for 10 min; 5 ul of a solution containing 50 nM tetramer was then added and the cells were incubated at room temperature for 30 min. The cells were washed in 2 to 3 ml of MACS buffer, resuspended in approximately 50 ul of a cocktail of fluorescently labelled surface marker-specific antibodies (see Reagents) and then placed on ice for 30 min. Subsequently, the cells were washed in 2 to 3 ml of MACS buffer, resuspended in 100 ul of MACS buffer and transferred to 96-well cluster tubes (Corning, Tewksbury, MA) for analysis.

### Interferon-gamma enzyme linked immunospot assays (IFN-γ ELISpot assays)

T cell responses were measured by IFN-γ ELISpot assay [10] using fresh PBMC. Peptides used for ELISpot assays were synthesized by Mimotopes, VIC, Australia (80% purity). The full length *P. falciparum* 3D7 AMA1 sequence (GenBank no. XM1347979) was covered by 153 15-mer peptides overlapping by 11 amino acids (aa) and grouped into 12 pools (Ap1-Ap12) each containing 10-13 peptides. The fact that six AMA1 peptide pools (Ap1, Ap3, Ap4, Ap8, Ap9 and Ap10) were immune-dominant has previously been reported using Ad-CA and Ad-C-immunized subjects[12], and, therefore, in the present study PBMC were stimulated for 36 hours with the immune-dominant AMA1 individual peptide pools using previously described methods[10]. A positive response was defined as fulfilling three criteria: that is (i) a significant difference (p = <0.05) between the average of the number of spot forming cells (sfc) in test wells and the average of those in negative control wells (Student’s two tailed *t*-test), (ii) at least a doubling of sfc in test wells relative to negative control wells, (iii) a difference of at least ten sfc between test and negative control wells, except where noted. Results shown in Table 1 are the magnitude of responses of each volunteer to the AMA1 peptide pool that contains the epitope expressed in the tetramer.

### FACS Data Acquisition and Analyses

**Figure 1:**
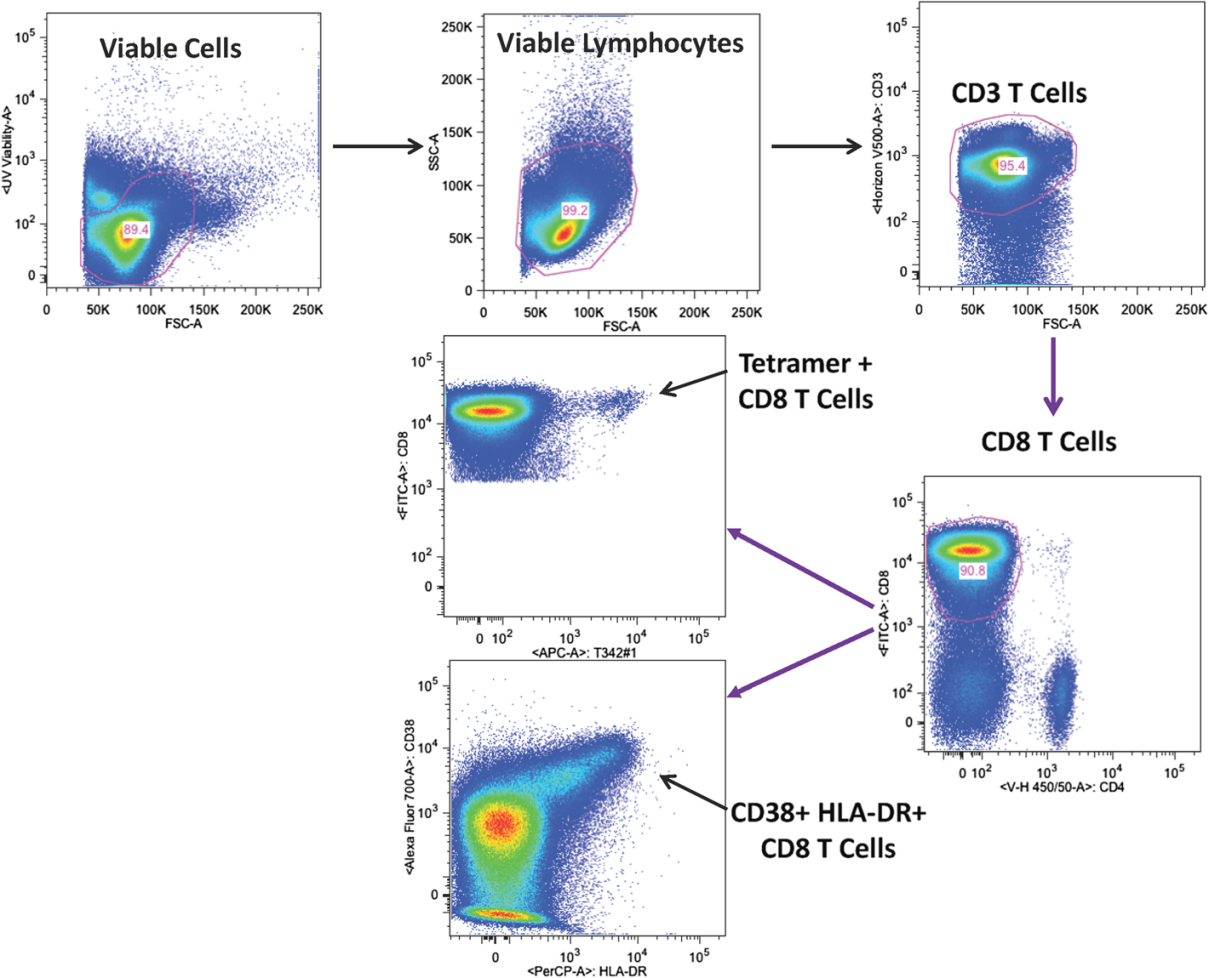
**Gating scheme.** PBMC were obtained from volunteers vaccinated with the NMRC-M3V-Ad-PfCA Vaccine (AdCA) and then CD8^+^ T cells were enriched by magnetic selection and labelled with cognate tetramers. Subsequently, cells were surfaced labelled using a single panel of antibodies for CD3^+^ T cells, CD8^+^ T cells, CD38^+^ cells and HLA-DR^+^ cells and then acquired on an LSR II flow cytometer. Data were analysed by Flow Jo and cells were gated as shown.

The cells were acquired on an LSR II flow cytometer (Becton Dickinson, Franklin Lakes, NJ) and the data were analysed using FlowJo (Tree Star) software. The gating scheme used in the analyses is shown in Figure 1.

## Results

### Direct *ex vivo* detection of malaria antigen-specific CD8^+^ T cells by MHC class I tetramer staining

**Figure 2:**
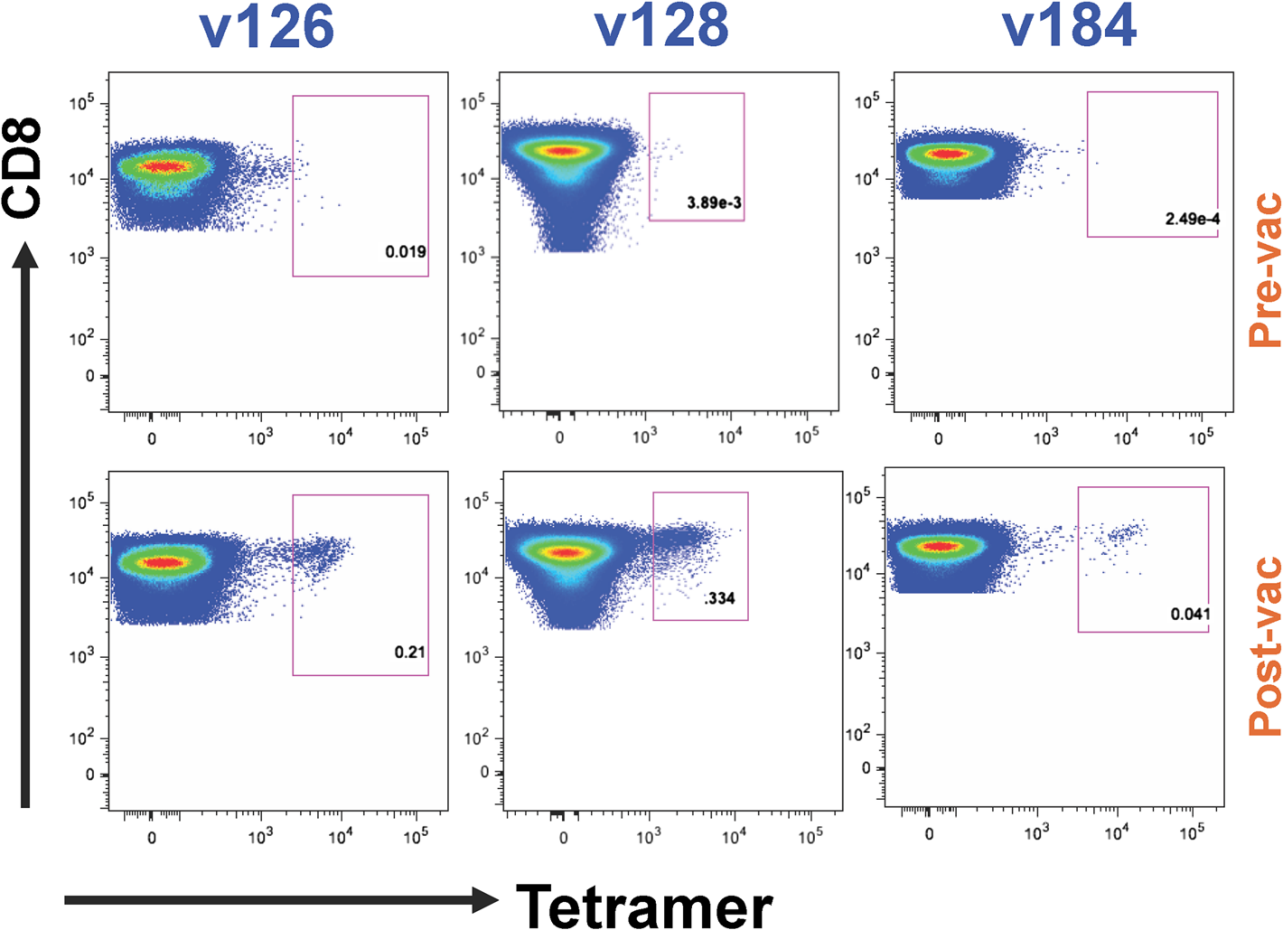
**Identification *ex vivo *of malaria antigen epitope-specific CD8**^**+ **^**T cells by cognate tetramer staining.** PBMC were obtained at pre {bleed 1}- and post {bleed 2 (day 22/23)}-vaccination. Enriched malaria antigen epitope-specific CD8^+^ T cells were labelled with cognate tetramers TLDEMRHFY:HLA-A*01:01(Vol. 126 and 184) and NEVVVKEEY:HLA-B*18:01(Vol. 128), surface labelled and then analysed as shown in Figure 1.

**Figure 3:**
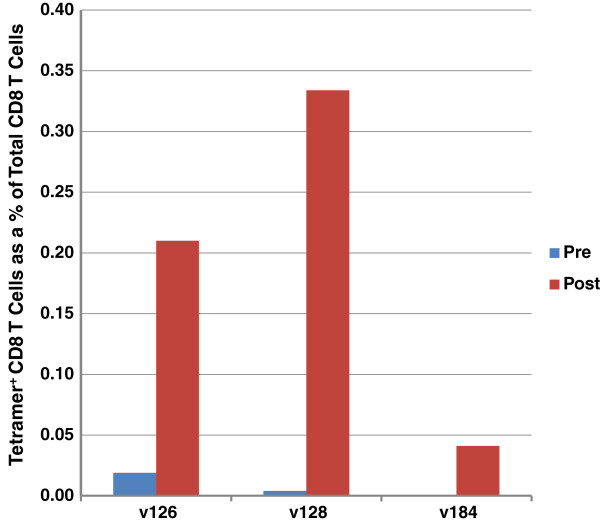
**Tetramer positive CD8**^**+ **^**T cells.** Tetramer positive CD8^+^ T cells are expressed as a percent of total CD8^+^ T cells.

PE- and APC-labelled tetramers consisting of predicted class I-restricted AMA-1 epitopes bound and presented by a subject’s MHC class I molecule (see Table 1) were tested for their capacity to label the volunteer’s CD8^+^ T cells. As seen in Figure 2, two of the tetramers: TLDEMRHFY: HLA-A*01:01 and NEVVVKEEY:HLA-B*18:01, were able to label tetramer-specific CD8^+^ T cells obtained at post but not pre-vaccination from two HLA A*01:01 volunteers (v126 and v184), and one HLA B*18:01 volunteer (v128), respectively. The frequency of the tetramer-specific CD8^+^ T cells was in the range of 0.041 to 0.33% of the total CD8^+^ T cell population (Figure 3). There was also some staining of the small number of residual CD4^+^T cells which was more apparent with the pre-vaccination cells and was likely non-specific in nature.

Four of the six cognate tetramers tested with volunteers 126 and 128 and all of the cognate tetramers tested with volunteers 119, 125 and 147 (see Table 1) did not label CD8^+^ T cells. The epitopes incorporated into the two tetramers that gave positive labelling of malaria antigen-specific CD8^+^ T cells were high affinity MHC class I binders (IC_50_ = 12-17 nM) and peptide pools containing these peptides also stimulated strong ELISpot responses. By contrast, two of the four non-staining tetramers contained peptide epitopes (RYKSHGKGY and TQKCEIFNV) predicted with low class I binding affinity (IC_50_ = 172-658 nM). Two of the other non-staining tetramers contained peptides (YEYPLHQEH and NEFPAIDLF) that were predicted with high class I binding affinity, but as compared to some of the other peptide pools shown in Table 1, these epitopes stimulated only relatively weak ELISpot responses.

### CD8^+^ T cells up-regulate activation markers following vaccination with the AdV-malaria vaccine

**Figure 4:**
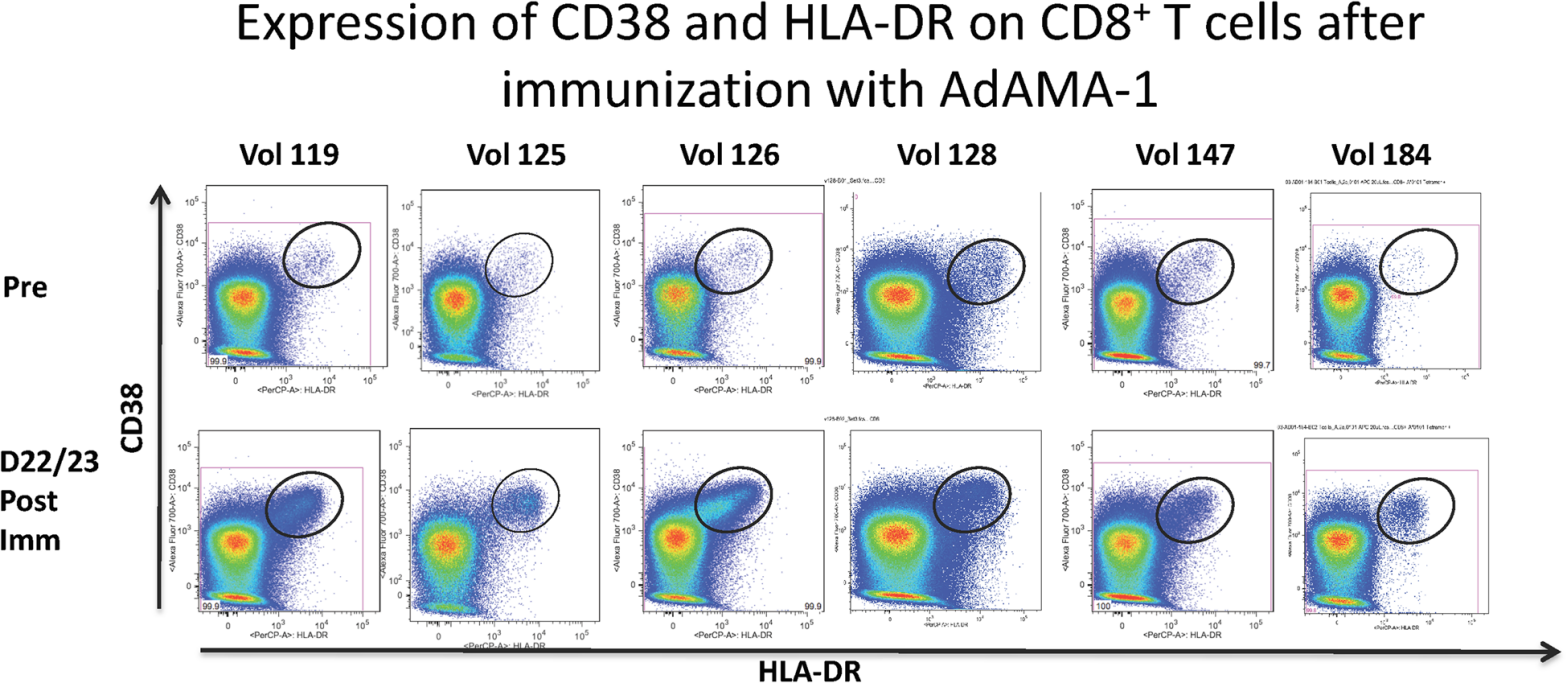
**Post-vaccination CD8**^**+ **^**T cells exhibit increased expression of early activation markers.** Malaria antigen epitope-specific CD8^+^ T cells obtained at pre- and post-vaccination were enriched by magnetic selection and then labelled exactly as described in the legend to Figure 1. Cells were then acquired on an LSR II flow cytometer.

As an alternative approach to detect malaria vaccine-reactive CD8^+^ T cells, experiments were carried out to determine whether CD8^+^ T cells responding to the malaria vaccine also up-regulated the expression of CD38^+^ and HLA-DR^hi^ early activation markers. As shown in Figure 4 and Table 2, in all six volunteers, CD8^+^ T cells obtained at 22/23 days post-vaccination exhibited a 3-17 fold increase in the frequency of cells expressing the {CD38 and HLA-DR^hi^}^+^ activation phenotype relative to those obtained at pre-vaccination.

**Table 2:** **% CD38 + HLA-DR**^**hi **^**CD8 + T cells obtained at pre-and post-vaccination**

**Volunteer**	**Pre-**	**Post-**	**Fold increase**
119	0.6	5.1	8.5
125	0.53	5.0	9.4
126	1	9.8	9.8
128	0.66	2	3
147	0.6	2.4	4
184	0.1	1.7	17

### CD38 and HLA-DR^hi^ serve as surrogate markers for malaria vaccine-specific CD8^+^ T cells obtained on day 22/23 post-vaccination

**Figure 5:**
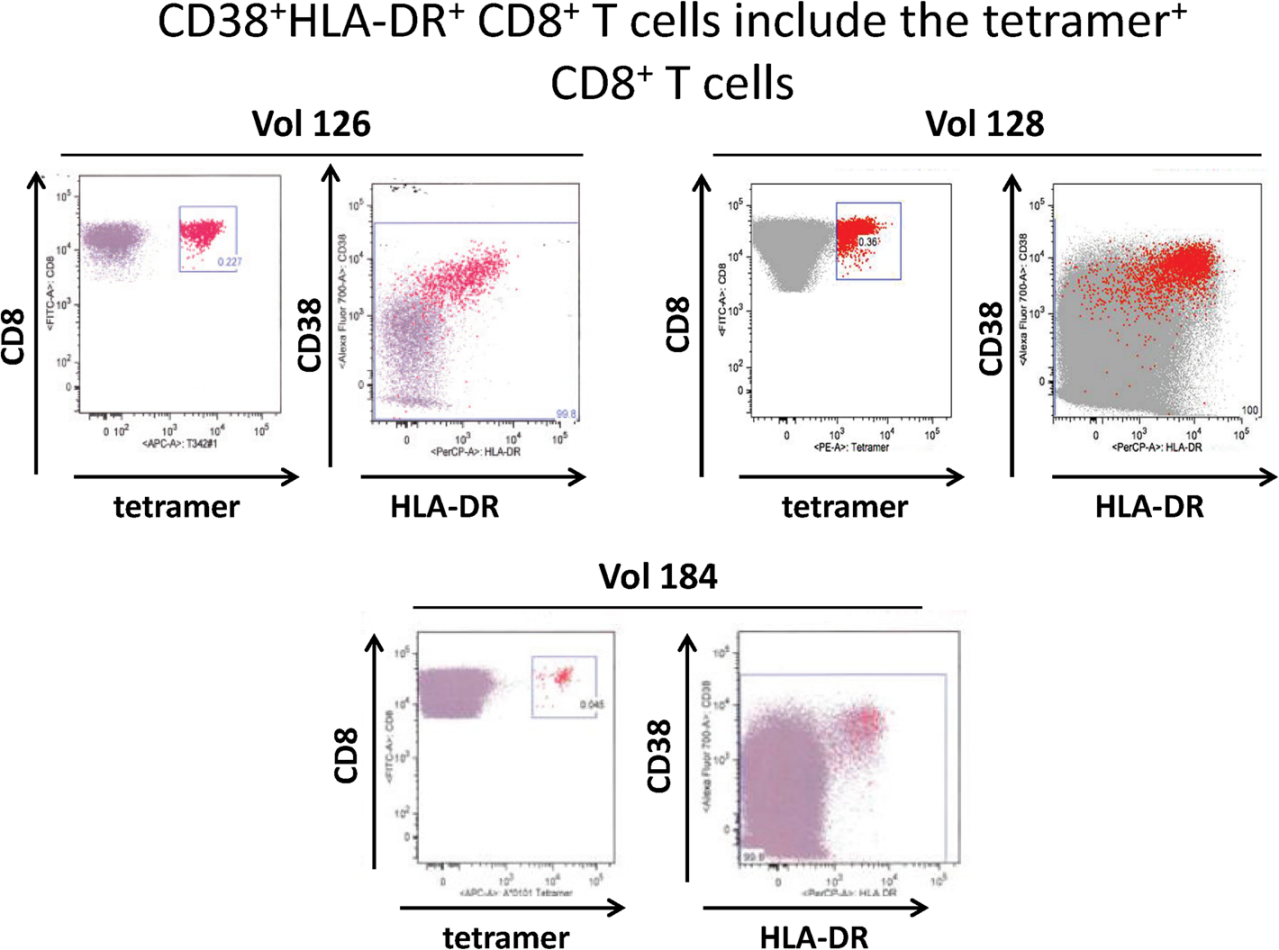
**Malaria tetramer-positive CD8**^**+ **^**T cells also express the {CD38**^**+ **^**HLA-DR**^**hi**^**} early activation phenotype.** Back-gating of malaria tetramer-positive CD8^+^ T cells (red dots) onto the total CD8^+^ T cells (grey dots) shows that the former are derived primarily from the {CD38^+^ HLA-DR^hi^}-positive sub-population.

To confirm that the CD8^+^ T cells detected by expression of activation markers were comprised, at least in part, of those identified by tetramer staining, further analyses were carried out to test for the coincidence of the two populations. As seen in Figure 5, back-gating the tetramer-positive cells obtained on day 22/23 demonstrated that the {CD38 and HLA-DR^hi^}^+^ CD8^+^ T cells encompassed the vast majority of the tetramer^+^ cells. Miller *et al.*[9] reported that vaccine-induced CD8^+^ T cells gradually lose their activation phenotype with time and this observation was confirmed with the demonstration that CD8^+^ T cells obtained from volunteer 128 at a much later time point (16 weeks post vaccination) could still be detected, albeit at lower frequency, by tetramer staining, but no longer expressed the {CD38 and HLA-DR^hi^}^+^ early activation phenotype (Additional file 1).

## Discussion

To establish methods to further verify the induction of malaria vaccine antigen-specific human CD8^+^ T cells and to determine the total magnitude of this CD8^+^ T cell response, a series of tetramers were prepared (Table 1), each consisting of one of three HLA-A* or one HLA-B* class I molecules and each presenting one of six distinct AMA-1 epitopes. Two of these tetramers, viz. TLDEMRHFY: HLA-A*01:01 and NEVVVKEEY:HLA-B*18:01, were able to directly label *ex vivo* and thereby identify and enumerate malaria antigen-specific CD8^+^ T cells in two AdCA-immunized HLA-A*01:01^+^ volunteers and one AdCA-immunized HLA-B*18:01^+^ volunteer, respectively. That these were vaccine-induced CD8^+^ T cells was further suggested by the fact that they were present at measurable frequency in the post but not pre-vaccination PBMC. Moreover, the CD8^+^ T cells were detected directly *ex vivo* without further *in vitro* manipulation and the majority of the cells expressed recent activation markers. Finally, the epitopes incorporated into the tetramers were identified by Class I binding algorithms and in previous studies [12] have been shown to stimulate T cell mediated cytokine production *in vitro* as detected by ELISpot assay (Table 1) and intracellular staining in matched research subjects.

The frequencies of the tetramer-labelled malaria vaccine epitope-specific CD8^+^ T cells ranged from 0.04 to 0.33% of total CD8^+^ T cells. Alanio *et al.*[15] have reported that the median frequency of naïve antigen-specific CD8^+^ T cells in humans is about 1 in 100,000, and if this was also true in the present system, then the malaria vaccine epitope-specific CD8^+^ T cells increased in frequency by approximately 40 to 330-fold following vaccination. Because the total CD8^+^ T cell population had been pre-enriched by magnetic sorting, the tetramer-labelled CD8^+^ T cells were readily visible and this implies that the technique should enable further phenotyping of these cells to determine whether they are SLECs or MPECs, as well as the sorting of the cells for polymerase chain reaction (PCR) and microarray analyses. In addition, such an approach should allow longitudinal analyses to follow the cells over time and to determine whether phenotypic changes occur following live sporozoite challenge.

To develop a method to potentially identify all vaccine-induced CD8^+^ T cells, without prior knowledge of their antigen specificity, the method of Miller *et al.*[9] and Querec *et al.*[16] was adopted, as these investigators had shown that all yellow fever virus-induced CD8^+^ T cells expressed the {CD38 and HLA-DR^hi^}^+^ early activation phenotype at two weeks post vaccination. Similar to these results, in the present study the AdCA-induced CD8^+^ T cells obtained at approximately three weeks post vaccination also manifested a 3 to 17 fold increase (Table 2) in the expression of the {CD38 and HLA-DR^hi^}^+^ early activation markers relative to that of the pre-immune cells. Significantly, as shown in Figure 5, the vast majority of the tetramer-labelled CD8^+^ T cells from the three positive volunteers also expressed the same activation phenotype, supporting the contention of Miller et al. that CD38 and HLA-DR^hi^ can potentially act as surrogate markers for all vaccine induced CD8^+^ T cells. The observation that CD8^+^ T cells obtained from volunteer 128 at a much later time point (16 weeks post vaccination) could still be detected, albeit at lower frequency, by tetramer staining but no longer expressed the {CD38 and HLA-DR^hi^}^+^ phenotype (Additional file 1) further supports the contention that cells expressing this phenotype are recently activated cells.

The lack of staining of CD8^+^ T cells from volunteers 126 and 128 by four of the six cognate tetramers tested and of CD8^+^ T cells from volunteers 119, 125 and 147 by all of the cognate tetramers tested (see Table 1) could have been due to several reasons including a low frequency of high affinity, tetramer-specific CD8^+^ T cell clones, lack of epitope generation and/or antigenic competition from other non-labelled vaccine epitopes. In support of the latter is the observation that tetramer^+^ CD8^+^ T cells comprised 2.1%, 16.5% and 2.5% of the total population of {CD38 and HLA-DR^hi^}^+^ CD8^+^ T cells from volunteers 126, 128 and 184, respectively. These percentages are in agreement with the notion that the activation markers detect all vaccine-induced CD8^+^ T cells which, in this present case, would also include CD8^+^ T cells specific for un-labelled malaria epitopes as well as the numerous potential epitopes processed from the viral vector proteins. It is likely, therefore, that only malaria antigen epitopes of high avidity for class I could become immune-dominant and able to overcome antigenic competition from other vaccine epitopes. In confirmation of this, it is noteworthy (see Table 1) that the epitopes incorporated into the two tetramers that gave positive labelling of malaria antigen-specific CD8^+^ T cells were high affinity MHC class I binders (IC50 = 12-17 nM) and peptide pools containing these peptides also stimulated strong ELISpot responses. The epitope NEVVVKEEY also exhibited a very high functional affinity and was able to stimulate positive ELISpot responses at concentrations below 10 nanograms/ml (data not shown). By contrast, two of the four non-staining tetramers contained peptide epitopes (RYKSHGKGY and TQKCEIFNV) predicted with low class I binding affinity (IC50 = 172-658 nM) and these epitopes, therefore, would be less likely to be immune-dominant and capable of competing with epitopes from other malaria and vector antigens. The other two non-staining tetramers contained peptides (YEYPLHQEH and NEFPAIDLF) that were predicted with high class I binding affinity but stimulated only relatively weak ELISpot responses, indicating concordance with the weak CD8^+^ T cell responses. These results suggest that peptide epitopes with both high-class I binding affinity and a capacity to stimulate strong ELISpot responses in the research subjects were optimal for guiding the selection of tetramers capable of labelling epitope specific CD8^+^ T cells generated by the viral vectored vaccine. The results also suggest that viral-vectored malaria vaccines may have to include high affinity MHC-binding malaria antigen epitopes that are capable of overcoming antigen competition in order to generate a CD8^+^ T cell response.

## Conclusions

The results, based on the limited samples available, demonstrate that cognate class I tetramers can identify *ex vivo* malaria vaccine antigen-specific CD8^+^ T cells. Especially if confirmed with additional data from an upcoming trial, it is expected that tetramer-labelling will permit the detailed assessment of antigen-specific CD8^+^ T cells to determine their frequency, cell surface phenotype (e.g.,. SLEC vs. MPEC) and transcription factor usage, potentially enabling the identification of immune correlates of vaccine-induced protection. Using research volunteers immunized with the Ad-CA vaccine, the present results also showed that expression of the {CD38 and HLA-DR^hi^}^+^ early activation phenotype was an excellent proxy for the identification all vaccine-induced CD8^+^ T cells. Whether, whole parasite or adjuvanted protein vaccines will also induce {CD38 and HLA-DR^hi^}^+^ CD8^+^ T cells populations reflective of the antigen-specific response will be the subject of future investigations.

## Competing interests

The authors declare that they have no competing interests.

## Authors’ contributions

RS, YK, BP and MS designed the experiments; SB and ASB prepared the tetramers; RS, GB, JE, MB, HG and JH performed the experiments; RS, GB, MB and MS analysed the data; RS, JE, CO, TR and MS wrote/revised the manuscript. All authors read and approved the final manuscript.

## Supplementary Material

Additional file 1 **PBMC were obtained from Vol. 128 at pre and 16 weeks post-vaccination.** Enriched CD8^+^ T cells were stained with cognate tetramers NEVVVKEEY:HLA-B*18:01 and then surface labelled for CD8^+^, CD38^+^ and HLA-DR^hi^. Cells were analysed as shown in Figure 1. Click here for file

## Disclaimer

JE, CO and TR were active duty military personnel at the time they contributed to this work; MS is a US Government employee. The work of these individuals was prepared as part of official government duties. Title 17 U.S.C. §105 provides that 'Copyright protection under this title is not available for any work of the United States Government.’ Title 17 U.S.C. §101 defines a U.S. Government work as a work prepared by a military service member or employee of the U.S. Government as part of that person’s official duties. The study protocol for the clinical trial presented in this manuscript was approved by the WRAIR and NMRC Institutional Review Boards, in compliance with all applicable Federal Regulations governing protection of human subjects. All study subjects gave written informed consent. The views expressed in this article are those of the authors and do not necessarily reflect the official policy or position of the Department of the Navy, the Department of the Army, the Department of Defense, or the U.S. Government.
