# Supplementary figures and images for: Ex vivo tetramer staining and cell surface phenotyping for early activation markers CD38 and HLA-DR to enumerate and characterize malaria antigen-specific CD8+ T-cells induced in human volunteers immunized with a Plasmodium falciparum adenovirus-vectored malaria vaccine expressing AMA1
Source: Malar J. 2013 Oct 29;12:376. doi: 10.1186/1475-2875-12-376 (PMC3819688; doi:10.1186/1475-2875-12-376)

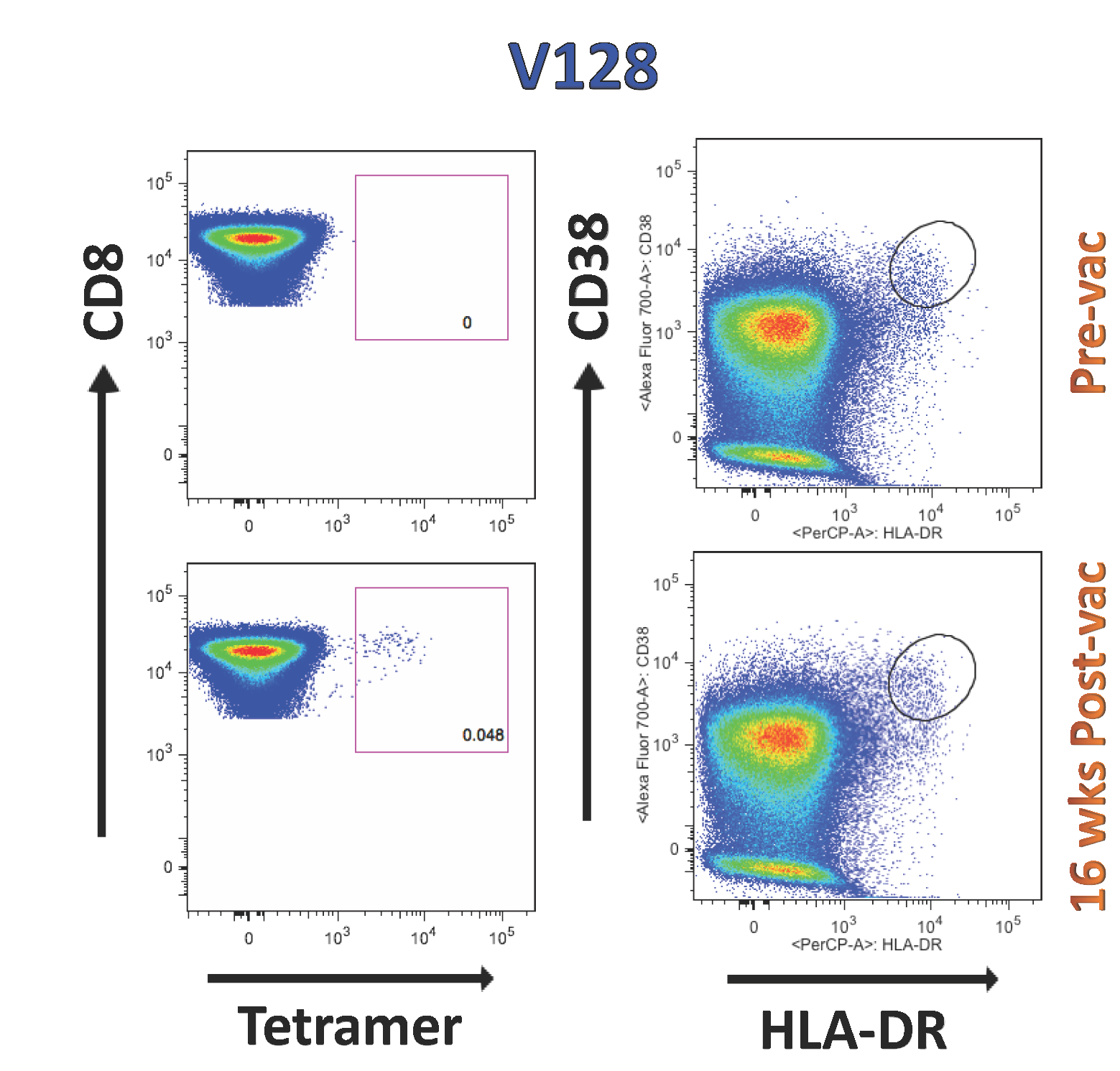

Supplement: Additional file 1 — PBMC were obtained from Vol. 128 at pre and 16 weeks post-vaccination. Enriched CD8+ T cells were stained with cognate tetramers NEVVVKEEY:HLA-B*18:01 and then surface labelled for CD8+, CD38+ and HLA-DRhi. Cells were analysed as shown in Figure 1. [file 1475-2875-12-376-S1.tiff]
